# Supplementary figures and images for: Investigating the potential of aggregated mobility indices for inferring public transport ridership changes
Source: PLoS One. 2024 Jan 5;19(1):e0296686. doi: 10.1371/journal.pone.0296686 (PMC10769062; doi:10.1371/journal.pone.0296686)

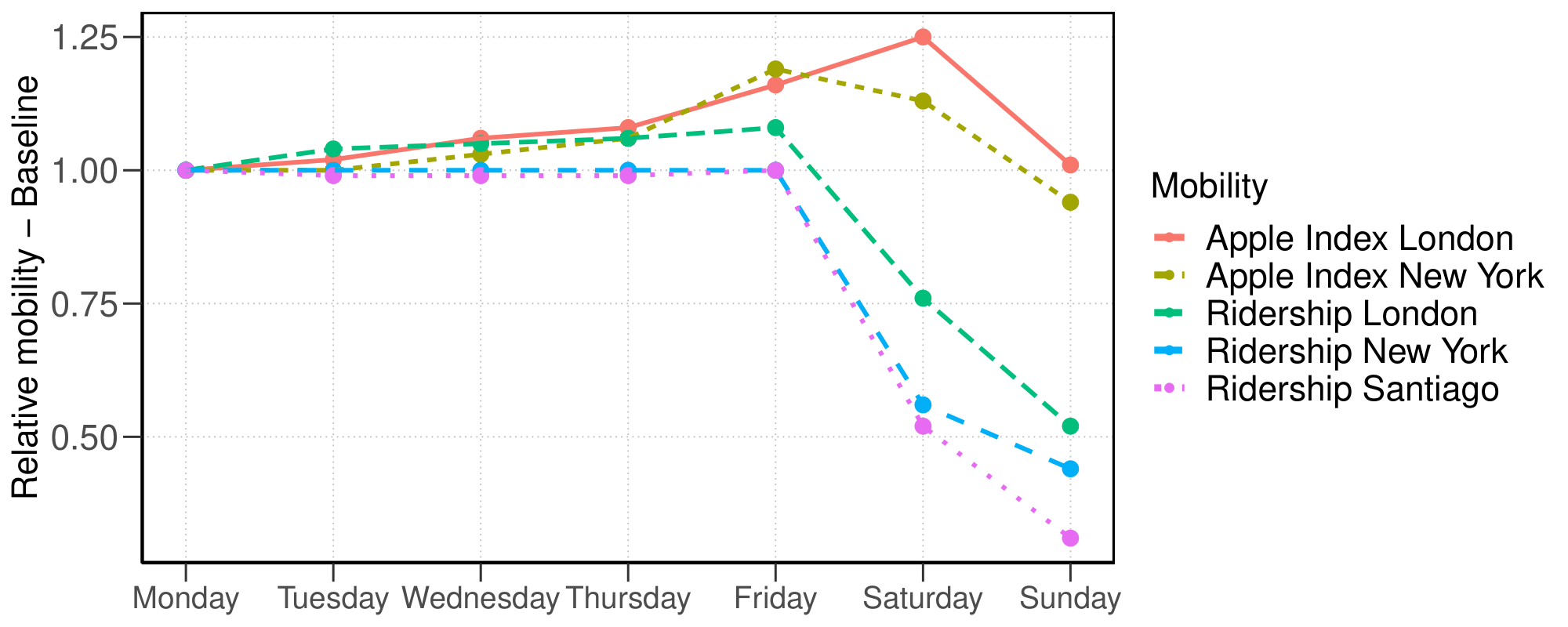

Supplement: S1 Fig — (TIF) [file pone.0296686.s001.tif]

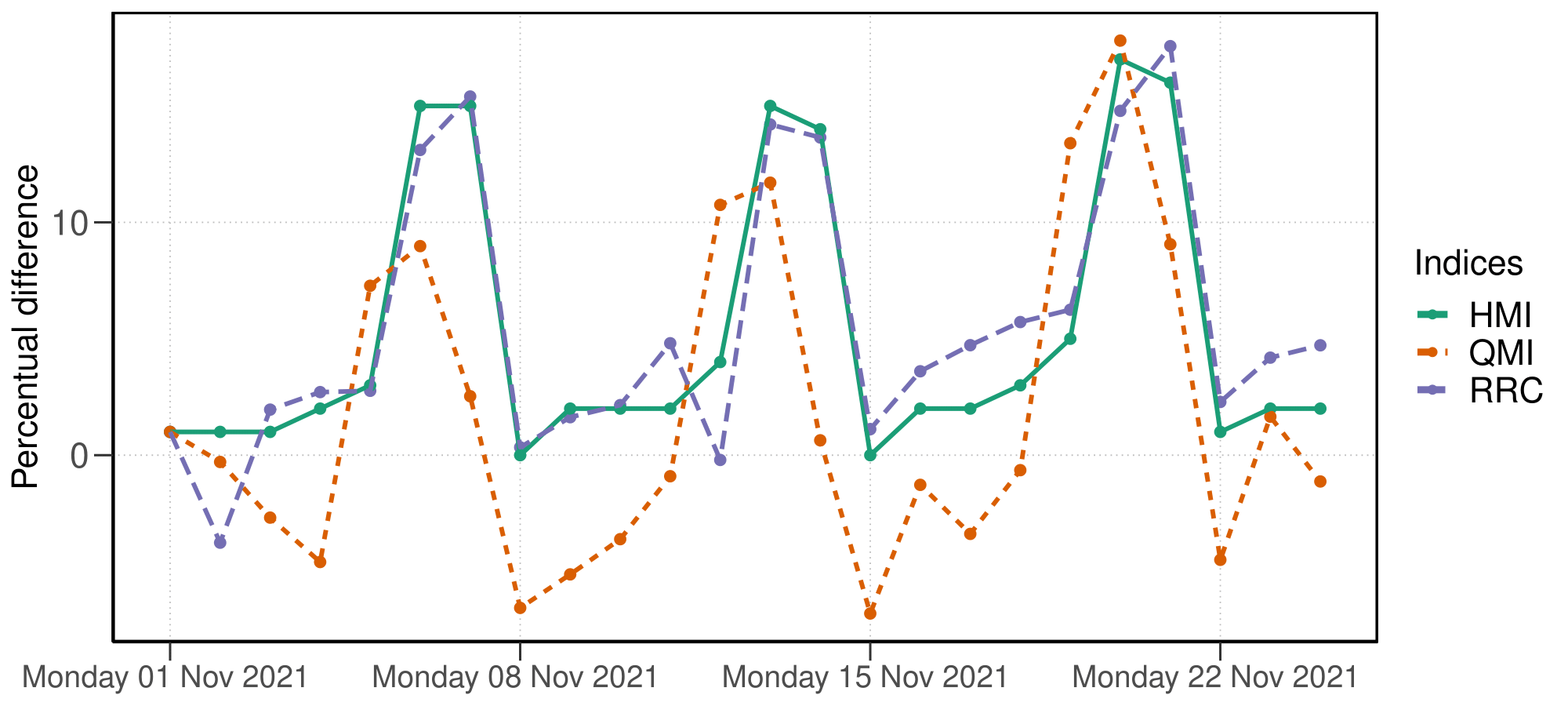

Supplement: S2 Fig — (TIF) [file pone.0296686.s002.tif]
